# The CD45 77C/G allele is not associated with myasthenia gravis - a reassessment of the potential role of CD45 in autoimmunity

**DOI:** 10.1186/1756-0500-3-292

**Published:** 2010-11-10

**Authors:** Ryan Ramanujam, Ritva Pirskanen, Lennart Hammarström

**Affiliations:** 1 Division of Clinical Immunology, Department of Laboratory Medicine, Karolinska Institutet at Karolinska University Hospital Huddinge, SE-141 86 Stockholm, Sweden; 2 Department of Neurology, Karolinska University Hospital Solna, SE-171 76 Stockholm, Sweden

## Abstract

**Background:**

The G allele of the CD45 77C/G SNP (rs17612648), which has previously been suggested to be associated with autoimmune disorders, was genotyped in 446 Swedish myasthenia gravis (MG) patients and 2303 matched controls.

**Results:**

There was no association between the polymorphism and patient group as a whole (*p *= 0.199), nor with clinical subgroups. Our results add to a growing number of studies unable to find association between the 77C/G polymorphism and autoimmune disorders. One control sample, from an adult blood donor, was homozygous for the G allele, yet negative for a panel of auto-antibodies, representing the first homozygous individual studied in this respect.

**Conclusions:**

The 77C/G mutation does not predispose to MG, and its role in autoimmunity may have to be re-evaluated.

## Background

Myasthenia gravis (MG) is an autoimmune disorder characterized by the presence of antibodies against the nicotine acetylcholine receptor on the muscle end-plate, thereby impairing transmission of nerve impulses to the muscle. MG occurs in 14/100,000 individuals in Sweden and patients commonly display thymic abnormalities such as thymoma and hyperplasia, where the former usually is associated with a severe disease [1]. Polymorphisms in several "classical" autoimmune genes have previously been shown to be associated with myasthenia gravis, including *IL-1*, *PTPN22 *and *TNF-α *[2]. Furthermore, an association has also been observed with the HLA haplotype A1, B8, DR3 [3-5], known to be linked to several "autoimmune" disorders [6-8].

CD45 (*PTPRC*), located on chromosome 1q31-32, is a receptor belonging to the protein tyrosine phosphatase family, consisting of molecules which have been shown to be involved in cell growth, differentiation and signaling. The receptor is heavily expressed on T-cells, where it comprises up to 10% of all surface proteins [9]. It has previously been shown to play a role in T-cell receptor signal transduction and activation as well as in thymic selection of T-cells, both important features in the development of autoimmunity [9], whereas a lack of CD45 expression results in severe immunodeficiency [10,11]. It undergoes complex, cell specific, alternative splicing to produce eight known isoforms. One isoform, containing exon 4 (CD45RA+), is expressed mainly by naïve T-cells, while an isoform with exons 4-6 spliced out (CD45RO+) is expressed by most memory T-cells [9]. The G allele of a low frequency single nucleotide polymorphism (SNP), 77C/G (rs17612648), has been reported to disrupt an exonic splicing silencer in exon 4, thereby leading to expression of higher levels of CD45RA on memory T-cells [12]. This, in turn, alters the T-cell activation threshold, providing a possible mechanism for development of autoimmunity [13].

CD45 shares homology and functional features with PTPN22, another protein member of the tyrosine phosphatase family. The latter contains a 1858C/T polymorphism (rs2476601) that has been shown to alter the T-cell activation threshold, due to an intracellular disruption of binding to the protein Csk [14]. This polymorphism has been strongly associated with many autoimmune disorders, including systemic sclerosis, rheumatoid arthritis (RA), systemic lupus erythematosus (SLE), MG, type I diabetes (TID) and multiple sclerosis (MS) [15-19]. Due to the similar role of CD45 in determining T-cell activation thresholds, a study investigating the association between the 77C/G polymorphism and MS was previously performed [20]. An association in three of four investigated populations was reported, thereby triggering a large number of replication studies.

This study was aimed at investigating association of this polymorphism with myasthenia gravis.

## Methods

### Patients and controls

Four hundred and sixty-six Swedish Caucasian MG patients and 2314 ethnically matched controls derived from anonymized adult blood donors (n = 1594) and dried blood spot samples from newborns (n = 720) from a population based study [21] were included in the study. The diagnosis of myasthenia gravis was made as described previously [1]. Antibodies against the acetylcholine receptor (AChR) were determined by radioimmunoassay [22], and testing for additional autoantibodies was performed using Bio Rad Bio-plex ANA and ANCA screens at the Karolinska University Hospital Laboratory. Immunoglobulin levels were determined by nephelometry at the Karolinska University Hospital Laboratory. Clinical information was documented by the primary physician over the course of treatment, and informed consent was given at the initial patient visit. Ethical permission was obtained from the Karolinska Institutet for use of patient and control materials.

### CD45 genotyping

Genotyping for the rs17612648 SNP in 466 MG samples and 2314 controls was performed using matrix-assisted laser desorption/ionization time-of-flight (MALDI-TOF) mass spectrometry [23] (SEQUENOM Inc., San Diego, California, USA) at the Mutation Analysis Facility of the Karolinska Institutet, Sweden. All samples that were found to be heterozygous or homozygous for the G allele were subsequently amplified and subjected to direct sequencing at Macrogen, South Korea, using the primers CTGGGAGGAGCATACATTTAGG and AGCACTAGCATTATCCAAAGAG, in order to verify the result.

### Statistical analysis

The Chi square test was used to compare the allelic frequency of CD45 in patients and controls. For all tests, a *p*-value below 0.05 was considered to indicate statistical significance. Power for the study was calculated using the "CaTS - Power Calculator for Two Stage Association Studies" http://www.sph.umich.edu/csg/abecasis/CaTS/[24].

### Patient subgrouping

Due to the complex nature of MG, which may contain several genetically distinct diseases exhibiting similar phenotypes, we stratified the patient material into subgroups based on the available clinical information. Patients were thus separated on the basis of sex, antibody status (anti-AChR positive or negative), thymic status (normal, hyperplasia or thymoma), disease severity (ocular, generalized or severe) as well as by age of onset. In the latter case, patients with age of disease onset less than 40 constituted the early onset group (EOMG), while those with an age of onset 50 or more were assigned to the late onset group (LOMG). Anti-AChR negative patients were defined as those who had never tested positive for anti-AChR antibodies, and had at least one negative test on record. We furthermore investigated possible association with known HLA biases within MG, specifically within the HLA B8, DR3 haplotype (EOMG) and the HLA B7 and DR2 alleles (LOMG) [25]. For each analysis, a Bonferroni correction was applied based on the number of independent subgroups created by the patient stratification.

## Results

Of the 466 genotyped MG samples, 20 were removed due to ambiguous readings, resulting in 446 appropriately typed samples. The number of control samples, after removing 11 samples with unsuccessful genotyping, was 2303.

The genotyping results are given in Table 1. Allelic variants of rs17612648 were not associated with myasthenia gravis in the patient group as a whole (*p *= 0.199), although the minor (G) allele appears slightly more frequently in MG patients than in controls (1.91% compared with 1.35%). Neither was any subgroup of MG associated with the SNP (*p *> 0.260, corrected), despite a slightly elevated minor allele frequency in most subgroups. Of all the patient subgroups, only the LOMG HLA B7 (0.91% MAF) and DR2 (0.81% MAF) subgroups had a lower frequency of the G allele than the control population (1.35% MAF), although, due to the low allele frequency, each group contained only one heterozygous case.

**Table 1:** Results of CD45 77C/G genotyping in myasthenia gravis patients and subgroups.

MG patients	n	G	C	MAF	*p*-val uncorrected	*p*-val corrected	OR	95% confidence interval for OR	**Power (%)**^**b**^
All patients	446	17	875	0.019	*0.199*	-	1.42	0.83-2.45	>99
Female	268	9	527	0.017	*0.532*	*1.000*	1.25	0.62-2.53	>99
Male	175	8	342	0.023	*0.151*	*0.302*	1.71	0.81-3.61	96
EOMG^a ^(age of onset ≤40)	208	9	407	0.022	*0.176*	*0.352*	1.62	0.80-3.28	98
LOMG^a ^(age of onset >50)	179	5	353	0.014	*0.936*	*1.000*	1.04	0.41-2.60	96
Hyperplasia	161	6	316	0.019	*0.442*	*1.000*	1.39	0.60-3.24	93
Thymoma	54	3	105	0.028	*0.207*	*0.621*	2.09	0.65-6.78	<70
Normal Thymus	60	2	118	0.017	*0.764*	*1.000*	1.24	0.30-5.14	<70
Ocular	42	1	83	0.012	*0.902*	*1.000*	0.88	0.12-6.44	<70
Generalized	289	13	565	0.022	*0.087*	*0.260*	1.69	0.92-3.09	>99
Severe disease	114	3	225	0.013	*0.969*	*1.000*	0.98	0.34-3.14	79
Anti-AChR ab. negative	54	3	105	0.028	*0.207*	*0.414*	2.09	0.65-6.78	<70
Anti-AChR ab. positive	389	14	764	0.018	*0.321*	*0.642*	1.34	0.75-2.41	>99
HLA B8, DR3 (EOMG)	85	3	167	0.018	*0.644*	*1.000*	1.32	0.41-4.24	<70
HLA-B7 (LOMG)	55	1	109	0.009	*0.693*	*1.000*	0.67	0.09-4.89	<70
HLA-DR2 (LOMG)	62	1	123	0.008	*0.605*	*1.000*	0.60	0.08-4.33	<70
Controls	2303	62	4544	0.013					

Allele frequencies for patients and subgroups are given as well as uncorrected and corrected *p*-values of the 77C/G polymorphism association with the disease. A Bonferroni correction was applied to subgroups based on the number of independent samples in each.

^a ^EOMG and LOMG patients were anti-AChR antibody positive without the presence of thymoma

^b ^The power of each group/subgroup comparison to detect the minimum allelic OR (4.53) from all studies reporting a significant association of the SNP to a disorder (Vogel, et. al., 2003, Table 2), at a significance level of 0.05.

**Figure 1:**
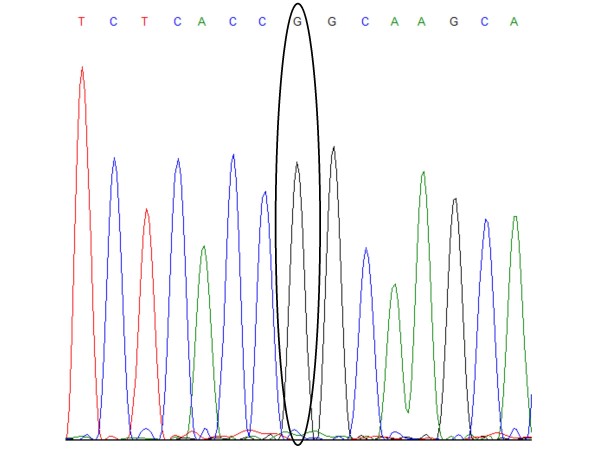
**Homozygous control sequencing chromatogram**. The sequencing trace for the homozygous control sample confirms that the individual contains the G/G genotype.

We furthermore observed a blood donor control sample homozygous for the G allele, which was confirmed by sequencing (Figure 1). Due to the presumed deleterious effect of homozygosity of this allele [26], a serum sample was tested in order to determine if autoantibodies were present. ANA antibodies (Anti-Nucleosome, Ribosomal P, RNP68, RNP A, Scl-70, Sm, SmRNP, SS-A(Ro52), SS-A(Ro60), SS-B, Centromere, Jo-1 and dsDNA) and ANCA antibodies (Anti-PR3, MPO and GBM) could, however, not be demonstrated. Serum immunoglobulin levels were also normal (IgM = 0.7 g/l, IgG = 9.7 g/l, IgA = 1.7 g/l). Due to restrictions in the ethical permission of the study, requesting anonymous control samples, material for additional analysis, including CD45 expression, could not be obtained.

## Discussion

The G allele of the CD45 77C/G polymorphism (rs17612648) was previously reported to be associated with MS in multiple patient cohorts, sparking interest in a possible common disease mechanism in related disorders. Table 2 presents an overview of the results of studies on CD45 77C/G association with autoimmunity to date. The compiled minor allele (G) frequency from these studies is approximately 1%; using this figure, our study has 80% power to detect allelic odds ratios greater than 2.5, far more sensitive than any study published to date which has reported significant association of the polymorphism to autoimmunity (Table 2).

**Table 2:** An overview of published findings investigating association of the 77C/G polymorphism with autoimmune disorders.

Study	Year	Population	Disease	Patients MAF(%) (n)	Controls MAF(%) (n)	OR	95% confidence interval for OR	Association
Jacobsen et. al. [20]	2000	Germany	MS	3.2 (219)	0.0 (189)	NA^†^	NA^†^	1.5 × 10^-4^
Jacobsen et. al. [20]	2000	Germany	MS	3.2 (108)	0.0 (114)	NA^†^	NA^†^	0.0058
Jacobsen et. al. [20]	2000	USA	MS	1.6 (122)	1.8 (244)	0.89	0.27-2.91	Not significant
Jacobsen et. al. [20]	2000	Germany	MS	3.3 (76)	0.4 (119)	8.06	0.93-69.68	0.0342
Barcellos et. al. [37]	2001	USA	MS	1.7 (450)	1.2 (253)	1.41	0.54-3.66	Not significant
Vorechovsky et. al. [38]	2001	Sweden	MS	1.5 (630)	1.4 (1044)	1.05	0.59-1.87	Not significant
Vorechovsky et. al. [38]	2001	Sweden	CVID	2.3 (44)	1.4 (1044)	1.60	0.38-6.78	Not significant
Vorechovsky et. al. [38]	2001	Sweden	IgAD	1.4 (148)	1.4 (1044)	0.94	0.33-2.69	Not significant
Vorechovsky et. al. [38]	2001	UK	CVID	1.5 (98)	0.9 (232)	1.79	0.40-8.06	Not significant
Vorechovsky et. al. [38]	2001	UK	IgAD	0.0 (17)	0.9 (232)	NA^†^	NA^†^	Not significant
Miterski et. al. [39]	2002	Germany	MS	0.8 (454)	1.4 (347)	0.53	0.20-1.40	Not significant
Wood et. al. [40]	2002	Germany	TID	0.2 (228)	1.3 (196)	0.17	0.02-1.46	Not significant
Wood et. al. [40]	2002	Germany	Graves	1.3 (297)	1.3 (196)	1.06	0.34-3.25	Not significant
Ballerini et. al.[27]	2002	Italy	MS	1.0 (194)	0.0 (222)	NA^†^	NA^†^	p = 0.02
Gomez-Lira et. al. [30]	2003	Italy	MS	1.2 (448)	0.9 (529)	1.30	0.55-3.08	Not significant
Schwinzer et. al. [31]	2003	Germany	SSc^a^	3.7 (67)	0.7 (205)	5.26	1.24-22.31	p = 0.029
Schwinzer et. al. [31]	2003	Germany	SLE	2.0 (98)	0.7 (205)	2.83	0.63-12.75	Not significant
Vogel et. al. [32]	2003	Germany	AIH^b^	3.2 (190)	0.7 (210)	4.53	1.27-16.19	p = 0.015
Tackenberg et. al. [35]	2003	Germany	MG	0.6 (78)	0.0 (303)	NA^†^	NA^†^	Not significant
Nicholas et. al. [41]	2003	UK	MS	2.6 (330)	2.0 (197)	1.28	0.55-2.98	Not significant
Thude et. al. [42]	2004	Germany	TID	1.2 (165)	1.1 (220)	1.07	0.28-4.01	Not significant
Cocco et. al.[43]	2004	Sardinia	MS	1.0 (246)	0.7 (226)	1.54	0.37-6-47	Not significant
Vyshkina et. al. [28]	2004	USA	MS	NA (176)^c^	NA (NA)	NA	NA	p = 0.0342^d^
Esteghamat et. al. [44]	2005	Iran	AIH^b^	0.0 (70)	0.4 (140)	NA^†^	NA^†^	Not significant
Kirsten et. al. [45]	2008	Germany	SSc^a^	0.6 (171)	1.4 (179)	0.42	0.08-2.16	Not significant
Pan-Hammarström et. al.[46]	2008	Sweden	IgAD	1.3 (232)	1.2 (913)	1.07	0.43-2.66	Not significant
Pan-Hammarström et. al.[46]	2008	Sweden	CVID	0.5 (91)	1.2 (913)	0.45	0.06-3.38	Not significant
Szvetko et. al. [34]	2009	Australia	MS	3.2 (155)	2.3 (171)	2.25	0.76-6.65	Not significant

^† ^The OR cannot be calculated for genotypes with some allele count of zero.

^a ^Systemic sclerosis

^b ^Autoimmune hepatitis

^c ^Number of families used to study transmission of G allele

^d ^Significance determined using 10,000 bootstrap samples in the software TRANSMIT version 2.5 after pedigree disequilibrium test failed to give a significant result

In nine follow up studies on association of the SNP with MS, only two have reported a significant association (*p *= 0.02 and *p *= 0.0342) [27,28]. In the latter study, the G allele was observed in 7 of 176 families and the pedigrees of these families were subsequently examined. Significance (*p *= 0.0342) was obtained after the pedigree disequilibrium test failed to give a significant result, after which 10,000 bootstrap samples simulated in the software TRANSMIT approximated significance tests instead of a χ^2 ^test. The lack of an accepted statistical approach, as well as an apparent MAF in cases (1.0-1.5%) similar to that in the Caucasian population (1-2%) [29], makes the results questionable. The lack of replication in MS is notable since the original study found an association in three out of four independent populations. An early meta-analysis of the first eight data sets determined that the range of results was due to heterogeneity between different studies (*p *= 0.01), and that exclusion of the first studies of Jacobsen, et. al. would remove heterogeneity (*p *= 0.23), resulting in a lack of association (*p *= 0.50) and an odds ratio of nearly 1 [30]. Of the 15 data sets from studies on other autoimmune disorders, only two have reported positive associations of the SNP to a disorder; systemic sclerosis (*p *= 0.029) [31] and autoimmune hepatitis (*p *= 0.015) [32].

Interestingly, homozygosity was observed in one of our control samples from an adult blood donor. To our knowledge, this is the first reported individual to be confirmed to be homozygous for the mutation. Tchilian et. al. did report a G homozygous anonymous thymus sample, but the patient was not available for further testing [33]. Svetko, et. al. also reported four G homozygous individuals (two samples in each of the MS and control groups), but did not conduct sequencing to confirm the finding, nor any additional investigations [34]. The overall MAF measured in the latter study (2.76%) is, however, higher than the aggregate reported for Caucasian samples (1-2%) [29], which indicates a possible overestimation of the G allele as does the fact that the genotype counts were not in Hardy Weinberg equilibrium (*p *< 0.05). Our control sample was negative for all tested auto-antibodies, which contradicts the previous assumption that individuals homozygous for the G mutation would be prone to autoimmunity.

A recent study investigated levels of CD45RO and CD45RA cell in German MG patients in which the rs17612648 SNP was suggested not to be associated with the disease. However, the number of cases and controls was too low (n = 78 and n = 303, respectively) to allow a solid conclusion (80% power to detect allelic OR greater than 5.6 at α = 0.05) [35]. In that study, ratios of CD45RO to CD45RA CD8+ T-cells were found to be significantly lower in patients with late onset MG (LOMG) as well as in T-cells in patients with thymoma. These differences suggest an alteration in CD45 expression independent of the rs17612648 SNP, and provide evidence that CD45 splicing may be regulated by other factors. In fact, Zilch et. al. have previously demonstrated that human/mouse somatic cell hybrids carrying only the mutant (G) allele are still able to generate CD45RO [36].

## Conclusions

Our results provide strong evidence for a lack of association of the rs17612648 SNP with MG. Furthermore, the presumed effect of the mutation in autoimmunity is not as strong as initially suggested, as most studies have failed to find an association. It is thus likely that the rs17612648 SNP is not the sole regulator of CD45 isoform expression, and that homozygosity for the mutation may result in neither propensity for autoimmunity, nor an absence of CD45RO expression.

## Competing interests

The authors declare that they have no competing interests.

## Authors' contributions

RR performed the statistical analyses, interpreted the results and drafted the manuscript. RP acquired patient material and analyzed clinical data. LH conceived of the experiments, interpreted the results and drafted the manuscript. All authors read and approved the manuscript.
